# Anterior Circulation Acute Ischemic Stroke in the Plateau of China: Risk Factors and Clinical Characteristics

**DOI:** 10.3389/fneur.2022.859616

**Published:** 2022-04-13

**Authors:** Yujia Yan, Xiqiang Zhang, Hecheng Ren, Xingwei An, Wanpeng Fan, Jingbo Liang, Ying Huang

**Affiliations:** ^1^Department of Neurosurgery, Tianjin University Huanhu Hospital, Tianjin, China; ^2^Academy of Medical Engineering and Translational Medicine, Tianjin University, Tianjin, China; ^3^Department of Neurosurgery, Third People Hospital of Xining City, Xining, China; ^4^Tianjin Center for Brain Science, Tianjin, China

**Keywords:** anterior circulation, plateau, altitude, clinical manifestations, acute ischemic stroke

## Abstract

**Background and Purpose:**

Acute ischemic stroke has a high incidence in the plateau of China. It has unique characteristics compared to the plains, and the specific relationship with altitude has not yet been appreciated. This study aimed to investigate the specificity of the plateau's anterior circulation acute ischemic stroke in China.

**Methods:**

To retrospectively collect clinical data of patients with first-episode acute ischemic stroke in the anterior circulation in Tianjin and Xining city. The differences in clinical presentation, laboratory, and imaging examinations were compared.

**Results:**

Patients at high altitudes showed a significant trend toward lower age (61.0 ± 10.2 vs. 64.8 ± 8.1, *P* = 0.010) and had a history of dyslipidemia, higher levels of inflammatory markers, erythrocytosis, and alcohol abuse. The main manifestations were higher diastolic blood pressure (85.5 ± 14.0 mmHg vs. 76.8 ± 11.6 mmHg, *P* < 0.001), triglycerides [2.0 (1.8) mmol/L vs. 1.3 (0.9) mmol/L, *P* < 0.001], CRP [4.7 (4.4) mg/L vs. 2.1 (1.9) mg/L, *P* < 0.001], homocysteine levels [14.5 (11.7) μmol/L vs. 11.2 (5.2) μmol/L, *P* < 0.001]; larger infarct volume [3.5 (4.8) cm^3^ vs. 9.0 (6.9) cm^3^, *P* < 0.001] and worse prognosis. Patients at high altitudes had higher atherosclerotic indexes in cIMT and plaque than those in plains.

**Conclusions:**

The natural habituation and genetic adaptation of people to the particular geo-climatic environment of the plateau have resulted in significant differences in disease characteristics. Patients with the anterior circulation acute ischemic stroke in the plateau show more unfavorable clinical manifestations and prognosis. This study provides a preliminary interpretation of the effects of altitude and suggests developing preventive and therapeutic protocol measures that are more appropriate for the plateau of China.

## Introduction

Stroke has a high rate of death and disability and is the second leading cause of death worldwide after coronary heart disease (1). Stroke is the leading cause of death in China (2). With the transformation of China's health care and demographic structure and the proportion of people over 65 years old has increased to 13.5%, the population is aging (3). The prevalence of stroke in China will continue to rise in the future, especially ischemic stroke, which severely impacts people's health in the acute phase (4).

The epidemiological distribution of ischemic stroke in China is distinctly geographical, with a high incidence in the plateau areas of northwestern China, such as Tibet and Qinghai (4, 5). An altitude above 1,500 m has an impact on human physiology. The unique climate, geographical environment, and customs have resulted in complex epidemiological features, pathogenic factors, and disease course characteristics (6, 7). Although the country has the largest plateau globally and a resident population of about 12 million (8), stroke in highland areas has not been extensively studied, and no separate guidelines for prevention and treatment have been developed.

In this study, we compared the clinical manifestations, imaging data, and prognosis of patients with anterior circulation acute ischemic stroke (AIS) in the highland and plain regions through a retrospective method to investigate the specificity and provide informative comments on the prevention and treatment of the disease.

## Patients and Methods

### Geographical Features

Patients with anterior circulation AIS are from Tianjin Huanhu Hospital and Xining Third People's Hospital. Both hospitals are provincial-level stroke treatment centers with similar pre-hospital transport, diagnosis, treatment, and care of patients.

Tianjin city is located in the North China Plain in eastern China, with a geographical location of 117°10′E, 38°34′N. The average altitude is 3.5 m, the average annual atmospheric pressure is 100.4 kpa. Xining city is located on the Tibetan Plateau in northwestern China, with a geographical location of 101°77′E, 36°62′N. The average altitude is 2,275 m, the average annual atmospheric pressure is 77.3 kpa. The ethnic groups in both places are mainly Han Chinese.

### Subjects

**Figure 1:**
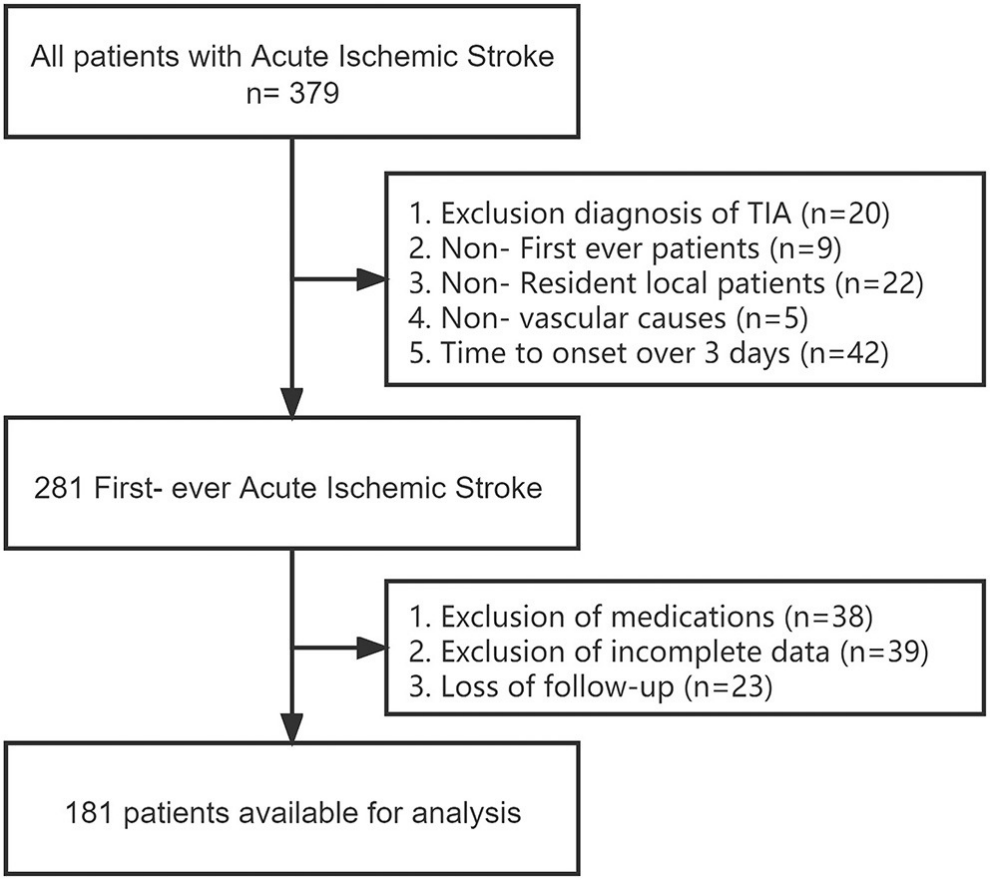
Flowchart of participants' selection.

We retrospectively selected a total of 379 patients diagnosed with first-episode anterior circulation AIS with a pre-onset mRS score of 0–1, >18 years of age and permanent residents (>10 years of residence) from May 2020 to 2021 in both regions electronic medical record systems. The diagnosis of AIS is based on the WHO definition, meeting clinical symptoms and examination criteria, and confirmed by computed tomography or magnetic resonance imaging of the brain. Exclusion criteria included: non-first episode, non-local patients; not caused by vascular diseases such as a tumor, trauma, hematologic disorders; previous use of drugs such as aspirin and statins; previous history of old cerebral hemorrhage/ischemia; transient ischemic attack (TIA), time from onset to hospital admission more than 3 days, and patients who were lost followed up for 90 days after discharge (missing, unreachable). A total of 181 patients were finally included in the study after screening with detailed and complete basic information, imaging examinations, and relevant laboratory test data (Figure 1).

### Data Collection and Assessment

Data collected included basic information on admission, vital signs (heart rate, blood pressure, oxygen, temperature), imaging examinations (MRI/CT/Carotid ultrasonography), routine blood and biochemical tests, coagulation, medical history (stroke, hypertension, diabetes, coronary artery disease), medication history (lipid-lowering, anticoagulant), National Institute of Health stroke scale (NHISS score). All data were selected from patients' first examination results within 24 h of admission. The follow-up endpoint was defined as day 90 after hospital discharge, and the day 90 mRS score was recorded as a prognostic indicator (dead within 90 days of follow-up were recorded as 6 points).

History of hypertension, coronary artery disease, and diabetes mellitus was based on previous and discharge diagnoses. Dyslipidemia was diagnosed based on past medical history and discharge diagnosis, with TC ≥ 6.2 mmol/L, TG ≥ 2.3 mmol/L, LDL-C ≥ 4.1 mmol/L, HDL-C ≤ 1.0 mmol/L meeting any of the diagnostic criteria (9, 10). Erythrocytosis is diagnosed by HB > 210 g/L in men; HB > 190 g/L in women (11). The bilateral mean or maximum CCA-IMT ≥ 1.0 mm was defined as an abnormal IMT; invasion of more than 50% of the arterial lumen thickness or showing a thickness >1.5 mm from the intimal to the epicardial interface is defined as plaque (12, 13).

### Imaging Process

**Figure 2:**
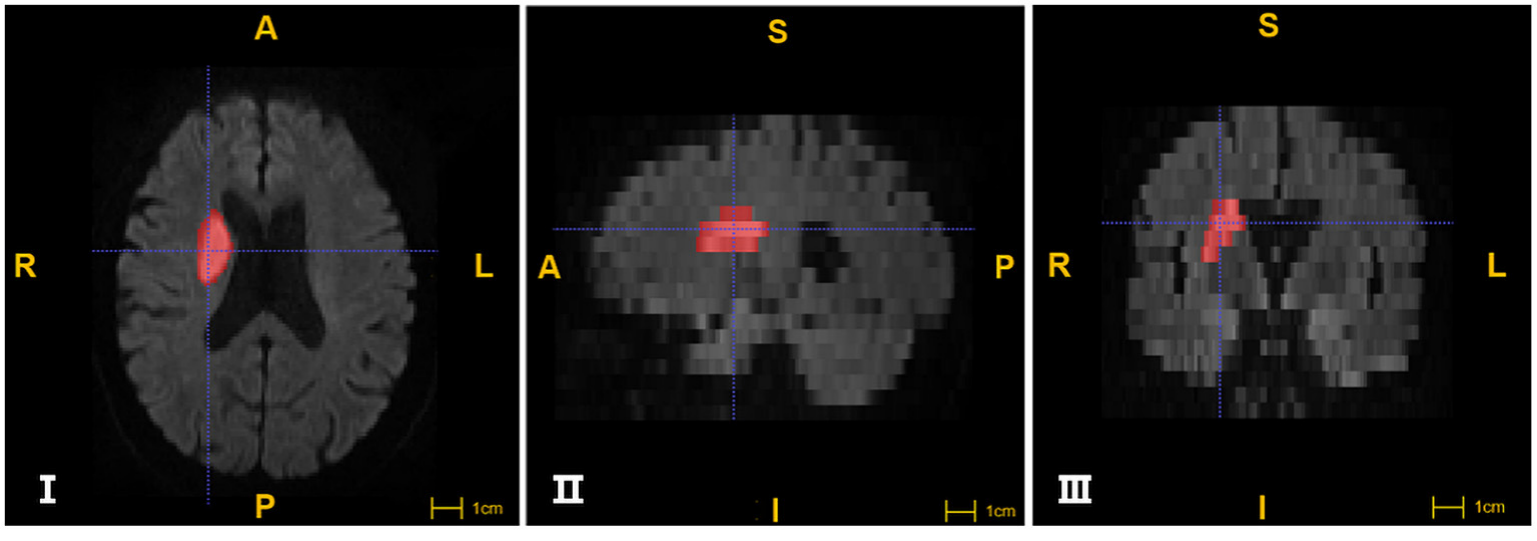
The segmentation of Ischemic lesions through ITK-SNAP software. **I, II, III** images were segmented from cross section, sagittal and coronal plane respectively. The areas in red are marked as infarcts (A: anterior; P: posterior; R: right; L: left; S:superior; I: inferior).

Head MRI images were processed using ITK-SNAP software (http://www.itksnap.org) (14). We used semi-automatic and manual forms to label infarct foci in cross-sectional, coronal and sagittal planes, respectively, and to perform volume calculations and 3D image imaging renderings (Figure 2).

### Statistical Analysis

All participants were classified into two groups according to altitude levels. Continuous variables were presented as mean ± SD or median (interquartile range). Categorical variables are expressed as frequency (percentage). SPSS v25.0 statistical software (IBM Corp.) was used for the data analysis. The continuous variables were analyzed by Student's *t*-test, the categorical variables were analyzed by Chi-square test and Fisher exact test. Non-normally distributed and non-parametric data using the Mann-Whitney U test and rank sum test. *P* < 0.05 was considered indicative of a statistically significant difference.

## Results

### Baseline Characteristics

One hundred and eighty one patients with anterior circulation AIS were included in this study: 133 from Tianjin and 48 from Xining. The basic characteristics and vital signs of the patients as shown in Table 1. Patients were admitted to the hospital for standard treatment (Intravenous Therapy, Endovascular Therapy) and subsequent rehabilitation.

**Table 1:** Baseline information and Vital signs of the patients at different altitudes.

	**Tianjin**	**Xining**	***P***
**Total (** ***n*** **)**	133	48	
**Altitudes (m)**	3.5	2,275	
Race	0.061		
Han (*n*, %)	130 (97.7%)	44 (91.7%)	
Other (*n*, %)	3 (2.3%)	4 (8.3%)	
**Age (years)**	64.8 ± 8.1	61.0 ± 10.2	0.010^*^
Gender	0.600		
Male (*n*, %)	86 (64.7%)	29 (60.4%)	
Female (*n*, %)	47 (35.3%)	19 (39.6%)	
Vital signs			
Respiratory rate (times/minute)	17 (1)	18 (2)	<0.001^*^
Heart rate (beats/minute)	69 (15)	75 (12)	0.001^*^
Body temperature (°C)	36.5 (0.3)	36.4 (0.4)	0.057
Oxyhemoglobin saturation (%)	99 (1)	97 (2)	<0.001^*^

^*^Continuous variables are expressed as mean ± SD or median (interquartile range). Categorical variables are expressed as frequency (percentage).

* *P < 0.05 was considered statistically significant*.

Patients in both regions were predominantly Han Chinese and male in terms of ethnicity and gender distribution. Patients at high altitudes had significantly higher respiratory rate and heart rate, while oxygen saturation was significantly lower, and there was no difference in body temperature between the two regions. In terms of age of onset, patients at high altitudes showed a significant trend toward a lower age (60.1 ± 10.2 vs. 64.8 ± 8.1, *P* = 0.010).

### Risk Factors and Laboratory Results

At high altitudes, the proportion of patients with coronary heart disease and diabetes was low, while dyslipidemia, homocysteine, CRP, erythrocytosis, hyperuricemia, and alcohol abuse was significantly higher. The proportion of patients with hypertension and a history of smoking was similar (Table 2).

**Table 2:** Risk factors with acute ischemic stroke at different altitudes.

**Variable (*n*, %)**	**Tianjin**	**Xining**	***P***
**Somking**	68 (51.2%)	25 (52.1%)	0.910
**Alcohol consumption**	47 (35.3%)	30 (62.5%)	0.001^*^
**Hypertension**	107 (80.5%)	34 (70.8%)	0.169
**Diabetes mellitus**	71 (53.4%)	15 (31.3%)	0.008^*^
Dyslipidemia	68 (51.1%)	39 (81.3%)	<0.001^*^
Hypertriglyceridemia	8 (6.0%)	20 (41.7%)	<0.001^*^
Hypercholesterolemia	5 (3.8%)	2 (4.2%)	0.173
**Coronary artery disease (CHD)**	66 (49.6%)	14 (29.2%)	0.014^*^
**Erythrocytosis**	0	7 (14.6%)	0.005^*^
**Hyperhomocysteinemia**	29 (21.8%)	24 (50.0%)	<0.001^*^
**Abnormal CCA-IMT**	30 (22.6%)	19 (39.6%)	0.023^*^
**Plaque**	44 (33.1%)	25 (52.1%)	0.020^*^

^*^Categorical variables are expressed as frequency (percentage).

* *P < 0.05 was considered statistically significant*.

Patients in the plateau had the most significant high diastolic blood pressure [85.5 ± 14.0 mmHg vs. 76.8 ± 11.6 mmHg, *P* < 0.001]. Dyslipidemia was characterized by hypertriglyceridemia [2.0 (1.8) mmol/L vs. 1.3 (0.9) mmol/L, *P* < 0.001]. HB/RBC levels were significantly higher in routine blood tests, and PLT levels were lower than in the plain; prolonged APTT and PT were demonstrated in coagulation function. In the vascular inflammatory indexes, homocysteine [14.5 (11.7) μmol/L vs. 11.2 (5.2) μmol/L, *P* < 0.001] and the blood C reactive protein (CRP) levels [4.7 (4.4) mg/L vs. 2.1 (1.9) mg/L, *P* < 0.001] were significantly higher in patients at high altitudes. Moreover, in carotid ultrasonography, the CCA-IMT [0.8 (0.5) mm vs. 0.7 (0.2) mm, *P* = 0.003] and the maximum thickness of plaque [2.6 (0.5) mm vs. 2.2 (0.8) mm, *P* = 0.002] were significantly higher in patients from high-altitude areas (Table 3).

**Table 3:** Laboratory results with acute ischemic stroke at different altitudes.

	**Tianjin**	**Xining**	***P***
Arterial blood pressure (mmHg)			
Systolic blood pressure	136.1 ± 21.0	137.9 ± 17.8	0.169
Diastolic blood pressure	76.8 ± 11.6	85.5 ± 14.0	<0.001^*^
Mean arterial pressure	96.5 ± 13.3	103.0 ± 14.7	0.006^*^
Glucose metabolism			
Glucose level (mmol/L)	7.0 (2.6)	6.0 (2.9)	0.003^*^
Glycosylated hemoglobin (%)	6.0 (2.1)	5.3 (1.3)	0.004^*^
Lipid Metabolism (mmol/L)			
TC	5.3 (1.3)	5.1 (1.0)	0.345
TG	1.3 (0.9)	2.0 (1.8)	<0.001^*^
LDL-C	3.2 (1.0)	2.8 (0.9)	0.001^*^
HDL-C	1.2 (0.3)	1.4 (0.4)	<0.001^*^
Blood cell			
RBC (*10^12^/L)	4.7 (0.6)	4.9 (1.1)	<0.001^*^
HB (g/L)	143 (16)	149 (17)	0.028^*^
PLT(*10^9^/L)	231 (70)	178 (75)	<0.001^*^
HCT/pcv (%)	0.4 (0.1)	0.5 (0.1)	0.002^*^
Blood coagulation			
APTT (s)	23.8 (4.3)	28.1 (4.4)	<0.001^*^
PT (s)	11.2 (0.8)	12.5 (1.4)	<0.001^*^
FIB (g/L)	2.8 (0.8)	2.9 (0.9)	0.918
TT (s)	17.6 (1.1)	17.9 (2.1)	0.070
INR	0.9 (0.1)	1.1 (0.1)	<0.001^*^
**CRP (mg/L)**	2.1 (1.9)	4.7 (4.4)	<0.001^*^
**Homocysteine level (μmol/L)**	11.2 (5.2)	14.5 (11.7)	<0.001^*^
**Uric acid level (μmol/L)**	307 (120)	333.5 (125)	0.016^*^
Carotid Artery Measurements (mm)			
Mean CCA-IMT	0.7 (0.2)	0.8 (0.5)	0.003^*^
Plaque maximum thickness	2.2 (0.8)	2.6 (0.5)	0.002^*^

^*^Continuous variables are expressed as mean ± SD or median (interquartile range).

* *P < 0.05 was considered statistically significant*.

### Volume of Infarction, NIHSS Score, and Prognosis Assessment

The infarct volumes and NIHSS scores of patients in both regions are shown in Table 4. Patients in the high-altitude region had a more severe neurological impairment and showed mainly consciousness level and facial palsy, sensory and speech dysfunction. Infarct volume increased significantly with increasing altitude, and patients with a grade of mild, moderate, and moderate-severe NIHSS scores all showed a trend toward larger infarct volume in patients at high altitudes. There was no difference in the distribution of ischemic lesion and vascular territory between the two regions.

**Table 4:** Differences in NIHSS score, Infarct volume, Ischemic description and mRS score at different altitudes.

	**Tianjin**	**Xining**	***P***
**NIHSS score**	7 (4)	10.5 (7)	<0.001^*^
Infarct volume (mm^3^)	3.5 (4.8)	9.0 (6.9)	<0.001^*^
Mild	2.1 (2.1)	7.9 (2.4)	0.001^*^
Moderate/moderate-severe	3.7 (5.0)	8.8 (7.4)	<0.001^*^
Severe	164.4 (–)	151.7 (99.9)	0.655
Ischemic lesion description (*n*, %)			
Cerebral cortex	36 (27.0%)	12 (25%)	0.781
Subcortical	97 (73.0%)	36 (75%)	
Basal ganglia	67 (50.3%)	26 (54.2%)	0.652
Vascular territory (*n*, %)			0.880
ICA	35 (26.3%)	13 (27.1%)	
ACA	6 (4.5%)	3 (6.3%)	
MCA	92 (69.2%)	32 (66.7%)	
**mRS score 0–2** (*n*, %)	106 (79.7%)	30 (62.5%)	0.018^*^

^*^The grade of NIHSS score: Mild (Score: 1–4), Moderate/ Moderate-Severe (Score: 5–20), Severe (Score: >20).

*Vascular territory: ICA (Internal carotid artery), ACA (Anterior cerebral artery), MCA (Middle cerebral artery)*.

^*^Continuous variables are expressed as median (interquartile range). Categorical variables are expressed as frequency (percentage).

* *P < 0.05 was considered statistically significant*.

We used the mRS score of 0–2 as the evaluation index of good prognosis, and it was seen that the proportion of patients with good prognosis was significantly higher at low altitude than at high altitude (70.8 vs. 88.0%, *P* = 0.035).

## Discussion

In the present study, we found that patients with anterior circulation AIS of the plateau region showed significant variability in clinical presentation, risk factors, laboratory tests, imaging features, and prognosis compared with those in the plains, as evidenced by abnormalities in substance metabolism, significant inflammatory responses, larger infarct volumes, more severe neurological symptoms, and poorer prognosis.

In the current studies of AIS, there are few detailed analyses in highland areas. Many factors in the altitude environment work together, such as low oxygen, low atmospheric pressure, low temperature, and intense ultraviolet radiation, among which the most significant is low-pressure hypoxia (15): As the altitude rises, the atmospheric pressure decreases, and the partial pressure of oxygen (PaO_2_) in the air also decreases. Oxygen is inhaled from the respiratory tract and passes through the alveoli, arterial blood, and cellular mitochondria. During the whole transportation process, the oxygen transfer volume decreases in a “waterfall” style due to the step-by-step decrease of PaO_2_, which eventually leads to oxygen availability in the intracellular mitochondria (18, 19). In addition, there is a combination of confounding factors such as ethnicity, lifestyle practices, health care awareness, and level of medical care that are difficult to eliminate. Therefore, our study selected areas with similar city sizes, economies, medical care, and ethnicity to reduce the confounding influences.

### Epidemiological Characteristics and Metabolic Changes

The relationship between altitude and the incidence of AIS has been reported to be highly controversial in different countries (20). Epidemiological surveys in China, India, and Kazakhstan showed a significantly higher risk of acute ischemic stroke at high altitudes (21, 22). In contrast, a Swiss survey showed the opposite result, with each 1,000 m increase in altitude reducing the risk of coronary heart disease and stroke by 22 and 12%, respectively (23). This study is based on the epidemiological findings of high incidence in the highlands of northwestern China and therefore does not discuss the differences in incidence by region.

In an international multicenter controlled study that included more than 22 countries, the significant risk factors for stroke in 90% of the world include the following six: hypertension, current smoking, abdominal obesity, diet structure, apolipoproteins, and physical activity (1). For plateau areas, due to their cold climate and traditional dietary habits, especially in nomadic Tibetan areas, the diet is characterized by high fat, high animal protein, high salt, less dietary fiber such as fruits and vegetables, and abuse of strong liquor, which significantly increase the risk of developing AIS (24–26).

#### Arterial Blood Pressure

In the relationship between arterial blood pressure and cerebrovascular accidents, diastolic blood pressure also has an independent impact on the risk of cardiovascular events and adverse outcomes (16). The importance of lowering diastolic blood pressure in preventing cardiovascular accidents is emphasized in the 2017 hypertension guidelines (27). Unlike systolic blood pressure, AIS in highland areas is characterized by increased diastolic blood pressure, which may be related to increased peripheral resistance due to increased erythrocyte and hemoglobin counts compensated by long-term hypoxia and increased blood viscosity. Therefore, the use of long-acting calcium antagonists that are highly selective for peripheral vascularity may have a better effect on blood pressure management in AIS prevention and blood pressure management in highland areas.

#### Blood Lipid Metabolism

Patients with AIS in the highlands have significantly more dyslipidemia, with high TG being the most significant. High TG may be related to a high-fat, high-animal-protein diet, with increased free fatty acids contributing to increased endogenous triglyceride synthesis in the liver (28, 29). In addition, the cold climate of the plateau also affects fat metabolism, and the body stores energy in the form of increased white fat through the storage of triglycerides (30). Elevated TG responds to an increase in very-low-density lipoprotein remnants, which can cross the arterial intima into the endothelium and cause recognition and clearance by macrophages, leading to endothelial dysfunction (31).

#### Blood Glucose Metabolism

The lower blood glucose levels in patients with AIS in the highlands may be related to the cold climate increasing the human metabolic rate and the expression of hypoxia-inducible transcription factor (HIF). Lee's study found that ten h of daily cold exposure at 19°C room temperature increased human insulin sensitivity, glucose uptake, and metabolic rate after 1 month (32). Genome-wide scans of highlanders exposed to chronic hypoxia showed that the HIF signaling pathway is highly expressed (33): HIF-1α acts in skeletal muscle to increase glucose utilization and glycolysis; HIF-2α acts mainly in the liver to inhibit hepatic gluconeogenesis, ultimately resulting in significantly lower fasting glucose levels in chronically exposed highlanders compared to the plains (34).

#### Erythrocytosis

Long-term chronic hypoxia stimulates the enhancement of bone marrow hematopoiesis and promotes the production of red blood cells and Hb to increase the oxygen-carrying capacity of blood and tissue oxygen supply. Therefore, among the pathological components of the thrombus, red thrombi with a predominant proportion of RBC and HB may be more frequent, suggesting that anticoagulation may be more effective. Therefore, in the treatment of AIS, it is essential to address the issues in the selection of antithrombotic drugs and the need to adjust the therapeutic dose in highland areas.

### Atherosclerosis and Inflammation

Carotid artery intima-media thickness (cIMT) and carotid plaque measures are commonly used to assess atherosclerosis (12, 13). We found that the cIMT and plaque burden were significantly higher in the highlands, as well as blood CRP and homocysteine levels. Atherosclerosis is a dynamic and active process, not just a disease of cholesterol or calcium salt accumulation. Inflammation plays a vital role in both atherosclerosis and thrombosis and coordinates disease progression and outcome (17, 35).

The hypoxic environment in the highlands induces high expression of the HIF pathway, and HIF-1α can promote the secretion of large amounts of inflammatory-related factors (such as IL-6/VEGF/IL-1β) from lipids, contributing to endothelial damage and aggravating the systemic and intravascular inflammatory response (36–38). Thus, in addition to the classic pathological lipid accumulation process, the disease process of AIS in highland areas may contribute more to thrombus formation through a thrombotic-inflammatory pathological response that promotes endothelial damage and inflammatory responses.

Due to the limitations of this article, most of the clinical information does not guarantee the accuracy of retrospective scoring, and we could not provide accurate TOAST sub-type retrospectively. We attempted to analyze further the pathogenesis based on the results of the article: The presence of larger infarcts usually excludes the small artery occlusion (SAO). Instead, it may suggest a cardioembolic stroke (39). The lower prevalence of coronary artery disease does not exclude cardiac embolism (CE) as a possible pathogenic factor. The relationship between CE and stroke appears to be more complex than a simple causal mechanism, not only for atrial fibrillation and ventricular thrombus but also due to other systemic and atrial factors, including systemic inflammation that may lead to atrial remodeling and subsequent atrial lesions (40, 41). Acampa et al. showed that inflammation is also a critical pathogenic factor in determining atrial cardiopathy and paroxysmal (and silent) atrial fibrillation (42). Inflammatory cytokines may alter the conduction properties of atrial cells and promote structural and electrical remodeling of the atria, which can further contribute to the development of CE (43). Moreover, the results of high blood CRP levels exhibited in the present study may confirm our hypothesis.

The use of statins facilitates lipid depletion in atherosclerosis and reduces inflammatory cell adhesion and monocyte recruitment by endothelial cells (17). The possibility of more active lipid-lowering therapy and the use of immune-targeted anti-inflammatory drugs to manage lipids in patients with the anterior circulation AIS of the plateau is vital for preventing cardiovascular and cerebrovascular accidents in the plateau and improving outcomes.

### Infarction and Prognosis

Comparison of infarct volumes and NHISS scores for the anterior circulation AIS between the two regions revealed that the plateau region had larger infarct volumes and more severe symptoms of neurological impairment, highlighted by impaired consciousness. There was significant altitudinal variability in infarct volume and degree of neurological impairment, the same as the currently available studies (5). All patients received standard therapy, and the 90-day well prognosis rate (mRS score 0–2) was significantly better in the plains than patients in the highlands. The large infarct size and severe functional impairment symptoms in the highlands may be related to the compensatory increase in RBC and HB levels, elevated blood viscosity, severe systemic and vascular inflammatory pathology, and the hypoxic environment that aggravates the cascade of neuronal cell damage after stroke, resulting in a double “hypoxic” injury and promoting the early development of the “ischemic penumbra” to the “infarct focus” (44–46).

Further grouping of NHISS scores revealed that plateau patients exhibiting mild, moderate, and moderate-severe AIS infarct volumes were significantly larger than those in the plains. In other words, the plateau population has improved the degree of tolerance of nerve cells and the body to hypoxia during the long-term habituation and genetic adaptation to the hypoxic environment. However, once AIS occurs, people in plateau areas need larger infarct volumes to get to the same clinical symptoms as plain patients, reflecting the decreased sensitivity of plateau people to AIS. The human body's sensitivity to the disease is a mechanism to avoid further damage, which is not conducive to the early detection and treatment of plateau AIS.

Moreover, homocysteine, a sulfur-containing amino acid formed during methionine metabolism, is an independent and graded predictor of cardiovascular and cerebrovascular events (including AIS/ myocardial infarction, etc.). The main mechanisms by which increased homocysteine leads to vascular events include impairment of vascular endothelial function, increased oxidative stress, abnormalities in lipid metabolism, and induction of thrombosis (47). Furthermore, the elevated blood homocysteine levels confirm the hypothesis above regarding the thrombotic component. Hyperhomocysteinemia may directly or indirectly determine alterations in atrial electrical conduction and favor atrial fibrillation occurrence with multiple mechanisms, increasing the risk of CE (43, 48).

Treatment with folic acid, vitamin B6, and B12 effectively lowers plasma homocysteine levels, and there is an optimistic view that lowering homocysteine levels can improve the prognosis of AIS (47, 49, 50). Therefore, for tertiary prevention and treatment of acute ischemic stroke in highland areas, we recommend a balanced dietary structure, avoiding a high-fat diet, increasing the intake of vegetables and fruits, and taking appropriate vitamin and folic acid supplements.

### Genetic Adaptation to the Geo-Climatic Environment

The plateau is one of the most challenging environments for human beings due to its extreme geographic climate, most notably to face the problem of hypoxia. According to Darwin's evolutionary mechanism, under the pressure of environmental and natural selection, humans and other life forms have changed their characteristics over a period. A short period of compensatory adjustment of function and structure is called acclimatization. Genetic adaptation is profoundly modified and reconstructed through genetic mutation, then consolidated through reproduction to future generations (51, 52).

It is interesting to note that the early adaptation process to hypoxia starts already in human infancy. In the early twentieth century, Barcroft showed by extrapolation from animal studies on sheep fetuses that human fetal PaO_2_ was between 2.5 and 3.5 kPa, equivalent to a hypoxic environment at 7,500 m above sea level. Thus, the environment in which the human fetus develops was described by him as the “Everest *in utero*” (53).

This study was limited by the human genetic studies involved, and no further genomic and proteomic characterization analysis was performed. However, the available findings argue that genetic adaptation of people to specific geo-climatic environments is a crucial factor in disease characteristics: The high-altitude hypoxic environment inhibits the expression of nitric oxide synthase (NOS), which induces acute high altitude pulmonary edema (HAPA) and pulmonary hypertension (54). Ahsan et al. determined that blood NO levels were significantly higher in the high-altitude population than at lower altitudes and that the wild-type GGbbAA TT combination of endothelial nitric oxide synthase (eNOS) genes was overexpressed. The GGbb combination was significantly associated with elevated NO, making the high-altitude population more tolerant of HAPE (55, 56). In addition, the results of genetic polymorphism studies on hemoglobin (HB), renin-angiotensin-aldosterone system (RAS), heat shock protein (HSP), and hypoxia inducible factor-1 (HIF-1) have shown the correlation between the genetic expression of specific genes and disease characteristics in highland populations acquired through long-term natural selection (33, 57, 58).

At present, in the field of plateau genomics, we have started to explore the long-standing problems in plateau and evolutionary biology. From the research content, the mechanism of plateau adaptation is altered by multiple molecular pathways to genetic factors. The specific gene regulation mechanisms of plateau adaptation are still unclear, and there is almost a gap in research related to plateau encephalopathy. Functional studies of genetic loci associated with genetic adaptation to the plateau are of great importance in the prevention and treatment of acute and chronic diseases of the plateau.

## Conclusion

In the anterior circulation AIS, patients in the highlands exhibited lower age, larger infarct volumes, more severe neurological symptoms, and poorer prognosis than the plains. The significant inflammatory response and atherosclerotic factors are essential for anti-inflammatory, lipid-lowering therapy in highland areas. We suggest that improving the dietary structure, reducing the high-fat diet, increasing the intake of folic acid and vitamins, limiting alcohol, strengthening the monitoring and management of blood pressure, blood lipids, and inflammatory indexes, and enhancing the awareness of health care have essential positive effects on the prevention of acute ischemic stroke in high altitude areas.

## Data Availability Statement

The raw data supporting the conclusions of this article will be made available by the authors, without undue reservation.

## Ethics Statement

The studies involving human participants were reviewed and approved by Tianjin University Huanhu Hospital. Written informed consent for participation was not required for this study in accordance with the national legislation and the institutional requirements.

## Author Contributions

YY, and XZ: study concept and design. YY, JL, and WF: data acquisition. YY, XZ, and HR: statistical analysis. HR, XA, and YH: interpretation of data. YY: manuscript drafting. JL and YH: review and editing. YH: supervision. All authors contributed to the article and approved the submitted version.

## Conflict of Interest

The authors declare that the research was conducted in the absence of any commercial or financial relationships that could be construed as a potential conflict of interest.

## Publisher's Note

All claims expressed in this article are solely those of the authors and do not necessarily represent those of their affiliated organizations, or those of the publisher, the editors and the reviewers. Any product that may be evaluated in this article, or claim that may be made by its manufacturer, is not guaranteed or endorsed by the publisher.
